# Immunoscore: a new possible approach for melanoma classification

**DOI:** 10.1186/2051-1426-2-S3-P193

**Published:** 2014-11-06

**Authors:** Mariaelena Capone, Gabriele Madonna, Noemi Sebastiao, Jean Bird, Fabrizio Ayala, Corrado Caracò, Gennaro Ciliberto, Bonnie La Fleur, Nicola Mozzillo, Gerardo Botti, Alisa Tubbs, Paolo Antonio Ascierto

**Affiliations:** 1 Istituto Nazionale Tumori, Fondazione "G Pascale", Naples, Italy; 2 Ventana Medical System, Tucson, AZ, USA

## Background

Increasing evidence has supported the hypothesis that cancer development is influenced by the host immune system. The number, type and location of the immune cells infiltrating the tumor microenvironment (TILs) may either limit or promote tumor progression [1]. These observations have led to the development of potential new scoring systems derived from the immune context in tissue and based on the identification and evaluation of specific lymphocyte populations. We focused on the potential prognostic value of CD3, CD8, CD20, and FOXP3 as an 'Immunoscore' for melanoma patients which would utilize widely accessible, standardized technology [2].

## Methods

We collected FFPE lymphadenectomies from 34 melanoma patients, analyzing a total of 150 lymphnodes. We have characterized the Immunescore by IHC expression of CD3, CD8, CD20 and Foxp3 (all Ventana Medical Systems). 3-4 micrometers serial tissue sections have been cut for H&E and stained with a multiplex of all markers including tumor marker for melanoma (S100). Tissue sections were stained using novel multiplex staining protocols on a VENTANA Benchmark instrument as well as serial staining. For each case, manual cell counts and regional annotation were taken. The number of positive cells has been evaluated by counting them in 5 peritumoral and 5 intratumoral non-overlapping fields using X400 magnification. The expression of each marker as well as combinations of markers have been matched with the most important clinical information of patients evaluating correlation with clinical outcome.

## Results

The data for each patient were summarized as a median expression across sampled nodes, and these values were then compared between relapse and no relapse groups. There were statistically significant differences in the peri/intra ratio for both CD3 and CD8, with the ratio being higher in no relapse patients compared to relapse patients for both proteins. These trending differences also seemed apparent for both FoxP3 and CD20, although our limited sample size limited some conclusions. We then hypothesized a high/low risk score which we now plan to validate on a larger melanoma cohort.

## Conclusion

Our results indicate that the CD3, CD8, CD20, and FoxP3 panel could be useful in defining the Immunscore, thanks to its prognostic value in high risk melanoma patients.

## Consent

Written informed consent was obtained from the patient for publication of this abstract and any accompanying images. A copy of the written consent is available for review by the Editor of this journal.
